# Content and Method of Information for Participants in Clinical Studies With Induced Pluripotent Stem Cells (iPSCs)

**DOI:** 10.3389/fcell.2021.627816

**Published:** 2021-04-28

**Authors:** Marcin Orzechowski, Maximilian Schochow, Michael Kühl, Florian Steger

**Affiliations:** ^1^Institute of the History, Philosophy and Ethics of Medicine, Ulm University, Ulm, Germany; ^2^Institute of Biochemistry and Molecular Biology, Ulm University, Ulm, Germany

**Keywords:** clinical translation, ethics, induced pluripotent stem cells, clinical research, informed consent

## Abstract

Research with induced pluripotent stem cells (iPSCs) involves specific ethical challenges, which should be addressed in the informed consent process. Up to now, little concern has been paid to the practice of information in iPSC-clinical studies. In order to fill this research gap, we have searched the documentation of the Research Ethics Committee at Ulm University from the years 2007 to 2019. In our previous research, we have identified 11 items for evaluation of the process of information in iPSC research. We used these items to analyze content and form of information provided for participants in the iPSC studies conducted at Ulm University and Ulm University Hospital in Germany. All analyzed studies provide general information regarding the study’s aim, method, and collection of donor’s personal data and specimen. The information for participants in these studies adheres to general guidelines for research involving human subjects; however, in several areas fails to take into account the specific nature of research with iPSCs. The majority of analyzed studies fail to provide information about possible individual consequences connected with genetic research, such as the possibility of re-identification of the donor or incidental findings acquired during research. Missing is also information about the possibility of future studies involving reproductive research or transplantation of cells and organs. The donor information process in all analyzed studies is conducted in form of the information sheet and oral information. The results of our research show that the process of informed consent in iPSC research should be updated as new developments emerge in this area. However, comprehension of information should not be jeopardized through information overload. Effective communication of essential information requires improved information methods tailored to the needs of participants, such as video animations, interactive consent modules or social media instruments.

## Introduction

Generation of mice cells with a developmental potential similar to human embryonic stem cells (hESCs) has been reported in 2006. Already one year later these cells, termed induced pluripotent stem cells (iPSCs), have been generated from human fibroblast (Shi et al., 2017). Before this date, pluripotent stem cells (PSC) from humans could only be derived from pre-implantation embryos. This development opened new fascinating horizons for regenerative medicine, especially concerning the modeling of disease development, drug testing for toxicity and efficacy, and therapeutic applications (Avior et al., 2016; Blau and Daley, 2019; Moradi et al., 2019). At the moment, iPSCs are used in a series of clinical studies involving retina transplants (Cyranoski, 2019), regeneration of heart muscle (Cyranoski, 2018), or growing of allogeneic iPSC-derived mesenchymal stem cells (Macdonald, 2019). Moreover, iPSCs have been widely hailed as an alternative to ethically disputed hESCs (de Miguel-Beriain, 2015; Zakrzewski et al., 2019).

Several specific features of iPSCs constitute their unique character (Lowenthal et al., 2012). iPSCs can be sourced from almost any kind of specimen, and used in potentially limitless rounds of derivation and differentiation. They carry a “genetic fingerprint” of the donor with an immeasurable amount of information that could lead to reidentification of the donor. iPSCs can also be source of incidental findings, i.e., information of the donor’s current or future diseases (Isasi et al., 2014). Moreover, iPSCs are immortal and provide a ubiquitous source of pluripotent material that can be stored for many years, widely shared, and used in a variety of research areas. These include transplantation into humans, creation of human-animal chimeras, growing human organs, creation of human clones or human gametes and embryos (Sugarman, 2008; Aalto-Setälä et al., 2009; Zarzeczny et al., 2009). Many further clinical applications of iPSCs are still not known at the moment and with certainty will be developed in the future.

The specific character of iPSCs defines ethical challenges related to clinical research in this area (Lo and Parham, 2009). These focus mainly on the scope and method of information for participants in such research (Greenberg et al., 2015). Our previous research identified four main thematic domains and 11 subdomains that should be included in donor information process in iPSC studies (Orzechowski et al., 2020). Because of the possibly endless longevity of derived biospecimen and the variety of potential uses for research and therapy, the provision of robust information and consent procedures becomes central from the ethical point of view. Such procedures increase participants’ autonomy and reduce insecurities regarding the transparency, methods, and goals of the research (Khan et al., 2014).

Although the issue of donor information and consent regarding iPSC-research has been widely discussed in the literature on the topic, up to now, only little attention has been paid to the question of donor information in practice (Lowenthal et al., 2012). In our research, we have examined in detail the content and form of information provided to participants in clinical studies with iPSCs conducted at Ulm University and Ulm University Hospital in Germany. The aim of the research was to provide answers to the following questions: 1. What was the specific content of the information provided to participants in these studies? 2. What procedures of information were used during the information process?

## Materials and Methods

### Methods

We have searched the documentation of the Research Ethics Committee at Ulm University from the years 2007 to 2019, that is, since the publication of the first research results on the possibility of utilization of human iPSCs. The search was conducted in the electronic repository of the Research Ethics Committee with the use of the following key-words in German and English: “induced pluripotent stem cells,” “pluripotent stem cells,” and “human induced pluripotent stem cells.” English keywords were used for the identification of possible multicenter studies conducted in cooperation with foreign research institutes. Additionally, we have examined the titles of all studies from this period, to check the completeness of the results. The search revealed eight studies with iPSCs conducted in this period. Through the repository of the Research Ethics Committee, we had access to all information materials provided for participants in the studies.

### Materials

In all identified studies, documentation for the Research Ethics Committee includes a detailed description of the proposed research, the method used in the research, and information for participants. The description of the research encompasses the specification of contact and acquisition of the participants. Furthermore, the documentation includes information sheets with a declaration of consent (DoC) and a contract of transfer (CoT) of biospecimen (also known as Material Transfer Agreement), which are to be signed by the participants in the study.

## Results

The search yielded eight results for studies using iPSCs, which were all included in our analysis. The examined studies included from 10 to 400 participants in patient and control groups. Seven of the studies included children or minors, either in the test or in the control group. All research projects focused on basic research with the use of iPSCs without the option of therapeutic use of the derivatives of differentiated cells. Five of the studies differentiated between information sheets for participants in the test group and participants in the control group. Analysis of the material for both groups shows however no differences in the specific iPSC-information content, only in expressions used to address each group. In studies that continued over several years, the information material has not been modified during the duration of the study.

Based on our earlier research (Orzechowski et al., 2020), information materials for participants in the studies were analyzed under consideration of four main thematic domains and 11 subdomains for evaluation of the process of information in iPSC research: 1. General information for the donors, 2. Information on storage of the cell lines and protection of privacy and confidentiality, 3. Information on research with the use of biospecimen, 4. Process of donor information (Table 1). These four main domains determine the following structure of this section.

**TABLE 1:** Domains of analysis of patient information in the identified studies.

Domains	Topic of information	Number of analyzed studies including the domain’s information
**I. General information for the donor**		
1. Study’s background information	The aim and method of the study	8 of 8 studies
	Collection of donor’s personal data	8 of 8 studies (only one study specifies what data will be collected)
	Biospecimen’s collection	8 of 8 studies specify the kind of donation, 3 studies specify the number of visits, 6 studies specify harm during the donation
	Contact information	8 of 8 studies (phone number, only in one case also email address)
	Voluntary nature of the study	8 of 8 studies
2. Genetic modification	Genetic modification of the biomaterial in the course of the study	1 of 8 studies
3. Commercial potential of the research	Property of body tissues	8 of 8 studies
	Financial compensation for the donor	8 of 8 studies
	Commercial result of the research	0 of 8 studies
	Individual and commercial interests of the researchers	0 of 8 studies
**II. Storage of the cell lines and protection of privacy and confidentiality**		
4. Storage of cells and cell lines	Information on the possibility of indefinite storage	6 of 8 studies
	Destruction of the biomaterial	8 of 8 studies (after 5 years–1 study, after 10 years–1 study, after 50 years–1 study, destruction only on donor’s request–4 studies, after the end of the research–1 study)
	Storage in repositories	8 of 8 studies (all in repositories of the research institution)
5. Protection of privacy and confidentiality	Association of biomaterial with particular individuals	2 of 8 studies
	Possibility of reidentification	2 of 8 studies
	Data sharing	7 of 8 studies
	Measures for the protection of the donor’s privacy	8 of 8 studies
6. Incidental findings	Possibility of acquisition of incidental medical findings	2 of 8 studies, in1 study only concerning hepatitis and HIV
	Procedures for the return of incidental findings	0 of 8 studies
**III. Research with the use of biospecimen**		
7. Future research	Use of biomaterial in other studies	8 of 8 studies
	Acquisition of the consent for other studies	0 of 8 studies
8. Reproductive research	Use of biomaterial for creation of gametes and embryos	4 of 8 studies
	Use of biomaterial for creation of human clones	4 of 8 studies
9. Transplantation of cells and organs	Use of biomaterial in regenerative medicine	4 of 8 studies
	Use of biomaterial for growing human organs	4 of 8 studies
10. Research on animals	Grafting of iPSCs into non-human animals	0 of 8 studies
	Creation of human-animal chimeras	4 of 8 studies
**IV. Process of donor information**		
11. Provision of information	Methods of donors information	in 8 of 8 studies through information sheet and oral information
12. Clarification of information	Evaluation of donor’s understanding of provided information	in 8 of 8 studies possibility to ask questions in 0 of 8 studies special protocol for evaluation of donor’s understanding

### General Information for the Donors

In all analyzed studies, participants are provided with general information encompassing the purpose and the method of the study, and a short outline of the individual medical or societal benefits of the research. Information that personal data will be collected for the purpose of the study is included in the DoC in all eight studies and additionally in the information sheet in four studies. However, only one study specifies which data will be collected—the name of the donor, disease, genetic data, and medical findings. In other studies, only general information about the collection of personal data and medical findings is included. All information sheets have information about the voluntariness of participation in the research and contact details of the supervisor of the research project or of a responsible researcher in form of a phone number or, in one case, an email address. Detailed information on the kind of biospecimen donation required, e.g., hair sample, is provided in all studies. Six studies declare that the process of derivation of body cells is painless and three studies specify the number of visits for the derivation of biospecimen. None of the analyzed studies provides donors with information about the unique nature of the human genome and possible individual consequences connected with genetic research. Only one study provides information about the genetic modification of the biomaterial in the course of the study.

Concerning information about the commercial potential of the research, all analyzed studies communicate that the donated biomaterial will become the property of the research institution and that no financial compensation for the donation will be provided. This information is provided in the CoT. Moreover, in all cases, the CoT provides a clause that commercial use of the transferred biomaterial is ruled out and that the material can only be used for scientific purposes. In five studies, this information is also mentioned in the information sheet. The materials do not include information about payment for donors from profits acquired from possible patents or products based on the results of the study, the commercial interests of the researchers and institutions conducting the study, or about sponsors of the study.

### Storage of the Cell Lines and Protection of Privacy and Confidentiality

Six of the analyzed studies include information about the possibility of indefinite storage of the donated biospecimen, in two cases this information is provided both in the information sheet and in the DoC for the study. CoT in all studies includes information that the donated biomaterial will be stored at the repository of the research institution; however, there is no specific information about the repository’s governance or review policies. With regard to the timeline of the storage, all studies communicate about the period for which the donation will be deposited. In four studies, it is 5, 10, and 50 years, or until the end of the research, respectively. The other four studies foresee an indefinite period of storage and the destruction of the donated biomaterial only on the donor’s personal and written request.

Regarding the protection of privacy and confidentiality, only two of the analyzed studies inform about the possibility of re-identification of the donor and association of the donor with medical data on the basis of donated cells. Data sharing is possible in the case of six studies, which inform that donated cells may only be shared with partners cooperating in this particular research project. The cells may be shared only in anonymized form with no personal data of the donor transferred to a collaborating institution. One study provides no information on whether the donor’s cells or data will be shared, and one study does not envisage sharing of the donor’s cells or data at all. In one study, the donors can specifically reject the transfer of their specimen to a collaborating institution. Specific formal clauses for the protection of donor’s privacy are included in all analyzed materials in the donor information sheet and in the DoC. According to these, all persons involved in carrying out the study are subjected to medical confidentiality and protection of individual data of the donors. The provided biospecimen is to be stored anonymously and the results of the study may only be published in an anonymized form.

Only one of the studies informs that in the case of future use of biomaterial there is a possibility of obtaining donor’s relevant medical information, such as predisposition to a certain disease. A clause for the return of such incidental findings is included in two of the studies. Additionally, one study informs that the specimen will be examined for HIV and Hepatitis B and C. However, none of the analyzed studies provide a specific protocol for the management and return of incidental findings.

### Research With the Use of Biospecimen

In all examined studies, the CoT states that the donation will be used for scientific purposes, especially for the particular study that the CoT refers to. This opens the possibility that the specimen will also be used in other studies as long as they have scientific character. However, there is no information whether consent for other studies will be subsequently acquired. In the case of the use of biomaterial for reproductive research, four studies provide no information on whether the material will be used for the creation of complex organisms, such as gametes, embryos, human clones, or human-animal chimeras. Four other studies inform that no complex organisms will be created during the research. Also, only four studies inform that the material will not be used in regenerative medicine or for growing human organs. None of the donor information materials contains information on whether iPSCs will be used for grafting into non-human animals.

### Process of Donor Information

All examined studies use information sheets for donor information. In three studies, these are directed to all participants. In other studies, information sheets are diversified and directed either to patients or to individuals from the control group. Seven studies include children or minors, either in the test or in the control group. However, specific information sheets for parents are included only in five studies. Moreover, only three studies include information sheets for minors or children. Information for minors is written in a more informal and simplified language than the information for adults. The information for children includes a graphic representation of the study method as well as short instruction about the donation of the biomaterial and the possibility of further inquiries about the research. One study involves the participation of mentally disabled persons; yet, no specific information sheet for this group is included in the documentation of the study.

In addition, information is provided in form of an individual talk with the donors. Responsible for oral information are researchers conducting the study. In some cases, such talk is conducted via phone, sometimes with the help of other communication means, i.e., Skype. After the call, potential donors received written information sheets, DoC, and contract of transfer. On the DoC stands the name of the researcher providing the information. Donors with their signature on the DoC confirm that they received explication about the content, method, risk, and goal of the study. They also certify that they had the opportunity to ask questions and that they received answers to these questions. No examined materials include any protocol for an assessment of information comprehension by the study participants.

## Discussion

So far, there exist numerous normative propositions regarding donor information in iPSC research. Our investigation examined donor information in practice in view of these normative statements. Previously, Lowenthal et al. (2012) analyzed the content of 25 iPSC-specific consent forms; yet, without providing explicit information about included content. To our knowledge, our research is the first analysis comparing in detail the proposed ethical norms with donor information in practice.

The normative literature on the topic highlights ethical concerns that may arise with regard to donor information in research with iPSCs. These concerns consider not only the involvement of human subjects but especially the specific character of the iPSC research. Numerous authors as well as guidelines issued by professional organizations address these issues (Aalto-Setälä et al., 2009; Lowenthal et al., 2012; Lomax et al., 2013; Greenberg et al., 2015; Daley et al., 2016; Moradi et al., 2019; Orzechowski et al., 2020). They recommend inclusion of particular information, which would safeguard donors from violation of their autonomy and allow them to make their own risk-benefit assessment regarding participation in iPSC studies.

### General Information for the Donors

Adequate information content should contain not only general frameworks established for research on human subjects but also specific information fundamental to the research in question (World Medical Association, 2020a). Our results show the extent to which these recommendations are implemented in practice in the case of iPSC studies conducted at Ulm University and Ulm University Hospital. Visible is that the examined studies follow such general frameworks but not always adhere to the specific need of iPSC research. Analyzed materials include information required by ethical principles for research involving human subjects as defined in the Declaration of Helsinki (World Medical Association, 2020a) and, through extension, by German Rules of Professional Practice for Doctors (Bundesaerztekammer, 2018). In all studies under investigation this information includes the general background, such as the aim and method of the study, risks, or details about the art of biospecimen collection. Included is also information about the voluntary nature of participation as well as about property rights of the biospecimen. However, missing is specific information on the nature of the human genome and about genetic modifications during the creation of iPSCs. As none of the examined studies foresees autologous intervention and transfer of the differentiated cell derivatives of iPSCs into donors’ bodies, missing information about genetic modification of iPSCs and cell lines does not have crucial importance for the donors.

Examined studies only partially provide information about the commercial potential of the research. Although one can find specific information about the property of donated biomaterial, missing is the issue of potential payments for donors in case of future products derived from the donation. Patent applications in the field of iPSC research need to be assessed from two standpoints: the rights of donors and their impact on efficiency in research, commercialization, and clinical translation of the technology (Kato et al., 2012). On the one hand, it has been argued that donors should be entitled to share the fruits of research they participate in, beyond access to new therapeutic methods (Hill, 2002). On the other hand, the proliferation of patent rights in this research area may lead to a situation, in which it will become inefficient and too expensive to exploit the technology in future studies (Bergman and Graff, 2007; Zarzeczny et al., 2009). Equal distribution of research rewards without impeding prospective studies and medical implementation still requires intensive discussion involving all stakeholders. However, the donors of biomaterial should have a clear view, already during the information process, about commercial applications of the research in which they participate and about possible financial benefits for them (Lomax et al., 2013; Greenberg et al., 2015). Similarly, information about the commercial interests of the researchers conducting the study, sponsoring institutions and companies could be important for donors and guide their decision about participation in the study (Lowenthal et al., 2012). This could improve the participant’s individual assessment of risks and benefits; however, at the same time, it should not hinder understanding of the information and consent process (Beskow et al., 2010).

### Storage of the Cell Lines and Protection of Privacy and Confidentiality

Within the domain of storage of biomaterial and protection of privacy and confidentiality, the examined studies provide information about the duration of the storage of biomaterial. The duration of storage significantly differs among the studies. Visible is that earlier studies defined a rather short period of storage, i.e., 5 or 10 years. With the progress of the research possibilities with iPSCs in recent years, also increased the declared duration of the period of storage. All studies provide information that biomaterial will be stored in a repository; however, they fail to include information about the repository’s governance and review policies. Provision of such information is crucial for donors, as it allows them to individually evaluate the safeguard measures for the protection of their privacy and it is designed to protect the rights of individuals, transparency, and inclusion (World Medical Association, 2020b).

Description of risk for privacy and confidentiality and measures to minimize these risks is a requisite in the case of studies with human biomaterial. Such information supports the autonomy of participants to make an individual decision about a tolerable amount of risk (Lowenthal et al., 2012). Especially in studies with iPSCs, the specific nature of the research should be taken into consideration (Toraldo et al., 2018). As cells, tissues, and iPSC lines carry a “genetic fingerprint” of the donor, the reidentification of the donor becomes possible on the basis of the genotype carried in the donated specimen (Lo and Parham, 2009; Isasi et al., 2014). In the examined studies, this information is stated only in two studies. All of the studies include specific clauses for the protection of participants’ privacy, which, however, do not acknowledge distinct measures of protection required in iPSC research.

Moreover, only two studies indicate the possibility of acquiring donor’s relevant medical information in form of incidental findings. Yet, none of the studies provides a specific protocol for informing participants about such findings. The possibility of identification of a disease or risk of a disease should be an object of particular consideration (Fabsitz et al., 2010; Crock et al., 2012). Protocols for return of incidental findings should be considered *a priori* and provided during the informed consent process (Lomax and Shepard, 2013; Kusunose et al., 2015).

### Research With the Use of Biospecimen

The possibility that the provided biospecimen will be used in various future studies with different methods and aims, constitutes crucial information for the donor. Although some donors might generally agree to use of their biomaterial in other studies than those envisioned at the moment of donation, they could object to some sensitive areas of research, such as research with reproductive purposes, transplantation of cells and organs, or research on animals (Aalto-Setälä et al., 2009; Lomax et al., 2013). Only four of the analyzed studies provide such information, although only indirectly in form of a declaration that the donated material will not be used for creation of complex organisms. Such broad formula encompasses areas of reproductive medicine, growing human organs for the purpose of regenerative medicine, or creation of human-animal chimeras. However, it fails to provide specific fields of possible research to which the donor can agree or reject. Reproductive research could include creation and destruction of human embryos or the creation of human clones—both of which could raise serious ethical objections for donors (Aalto-Setälä et al., 2009; Zarzeczny et al., 2009; Dasgupta et al., 2014). Use of iPSCs in therapeutic or regenerative medicine involves the transfer of a donor’s genetic code into the body of another individual. This could also pose an ethical dilemma for a prospective donor (Lowenthal et al., 2012). Similarly, ethical concerns for donors might arise in case of research involving grafting iPSCs into animals or creation of human-animal chimeras. Though these applications can be invaluable for research on the treatment of some diseases, such as Parkinson, donors might oppose such uses because of their religious convictions or outright opposition to research on animals (Sugarman, 2008; Aalto-Setälä et al., 2009; Nöthling-Slabbert and Pepper, 2015).

The analyzed studies leave open the possibility of future scientific studies without clearly defining participants’ agreement to these or the way of obtaining future consent. This could lead to limiting donors’ right to an individual decision. In case of an agreement to future studies, central becomes the issue of balancing participants’ rights to an autonomous decision and sustaining the process of scientific progress. Here, it needs to be considered whether donors should provide a blanket consent for the use of their biomaterial for all future studies or remain in sustained interaction with the researchers regarding new research aims. The first option allows to avoid delays and administrative burdens and to increase the effectiveness of the research (Lomax and Peckman, 2012; Kass et al., 2013). However, it violates the donor’s autonomy, which depends on individual convictions, is not static, and can change over longer periods of time. Therefore, participants should be enabled to prospectively control the use of their specimen through other forms of consent, e.g., through personalized, secure, and digital communication interface that provides researchers with a possibility of continuous contact with donors (Kaye et al., 2015). Such a dynamic consent approach brings benefits for both sides: researchers and donors. Researchers can easily approach donors regarding new research initiatives and receive a response promptly. Donors can tailor and manage their consent preferences.

### Process of Donor Information

With regard to the process of information, all analyzed studies combine the use of information sheets with the individual provision of information through personal contact with the participants. Such an approach can provide better results than the sole use of written information (Kusunose et al., 2015). However, sometimes individual contact occurs via phone, which can put certain restraints on the information process and lead to a poor understanding of the provided information. Because of the complexity of information and specific nature of the iPSCs research, proposed are alternative approaches in this area (McCaughey et al., 2016a, b). It has been shown that the use of computer animations, videos, or group discussions explaining the nature of the research can lead to improvement of the information process and to a better understanding of the aim, methods, and nature of the proposed research (Liu and Scott, 2014). Moreover, central to the process of information should be an assessment of the donor’s level of understanding. Oftentimes, provided information can be incomprehensible or misunderstood. Therefore, the information should be delivered in a way that is tailored to the perception abilities of the donors (Kusunose et al., 2015). This is especially important in research with vulnerable groups, such as children or mentally handicapped (Liu and Scott, 2014). In the case of all examined studies, the participants are explicitly invited to ask questions, which positively influence the comprehension of information and assessment of understanding. However, although seven of the analyzed studies involve research with children and minors, only four give children specific information and only one study’s information sheet specifically aims minors. Our analysis shows the need for improvement in this area. Creative and applicable methods for assessing capacity to consent and for information disclosure tailored specially for the needs of vulnerable groups can promote decision making. At the same time, these methods allow the involvement of vulnerable groups in the decision-making process and respect their autonomy. Possible improvements could involve the use of educational videos, computer animations, or social media with information specifically tailored for non-adult participants. The benefit of using social media is an increased number of interactions with researchers and the provision of information specific to participant needs (Topolevec-Vranic and Natarajan, 2016; Gelinas et al., 2017). Yet, also disadvantages of this approach should be considered, such as information overload or possible security breaches (Househ et al., 2018).

The findings from this research need to be considered in light of its limitations. Our analysis consists of a sample of eight studies conducted at two research institutions in Germany. Although the size of the sample is too small to provide generalized results with regard to other institutions or countries, it allows us a better understanding of the practice in the field of research with iPSCs and compliance with the ethical norms guiding such research. The size of the sample provides also an advantage—it allows detailed inspection of research protocols submitted to the Research Ethics Committee at Ulm University. To our knowledge, up to now, only one other investigation of such compliance with ethical guidance has been attempted (Lowenthal et al., 2012). Our analysis considers the newest state of research in this area as well as guidelines issued by international bodies, such as International Society for Stem Cell Research (ISSCR) (Kato et al., 2012; Daley et al., 2016).

In conclusion, the unique character of research with iPSCs requires adherence to general rules of research on human subjects and also special attention to several other ethical aspects of patient information. Reflected should be a broad approach to consent, which includes the provision of specific information that allows study participants to individually evaluate the risks and benefits of partaking in the study. Although analyzed studies observe general rules of patient information, crucial disclosure of specific risks to patient’s privacy or ethically objectionable areas of future research are missing. Such shortcomings need to be alleviated in order to ensure the donor’s autonomy. Important is that the process of informed consent keeps pace with current developments in iPSCs research. However, comprehension of information should not be endangered through the provision of excessive details, which would decrease the general understanding of information and demotivate patients to participate in the study. Here, a balance between the donor’s rights and researchers’ interests needs to be achieved. Alternative methods of information, tailored to adult and non-adult participants, such as video animations, interactive consent modules or social media groups can support the comprehension of information materials without decreasing the extent of substantial disclosure.

## Data Availability Statement

The original contributions presented in the study are included in the article/supplementary material, further inquiries can be directed to the corresponding author/s.

## Author Contributions

All authors listed have made a substantial, direct and intellectual contribution to the work, and approved it for publication.

## Conflict of Interest

The authors declare that the research was conducted in the absence of any commercial or financial relationships that could be construed as a potential conflict of interest.

## Abbreviations

CoT: Contract of Transfer
DoC: Declaration of Consent
hESC: Human embryonic stem cell
HIV: Human immunodeficiency viruses
iPSC: Induced pluripotent stem cell
ISSCR: International society for stem cell research.
